# Fetal gut cell-like differentiation in esophageal adenocarcinoma defines a rare tumor subtype with therapeutically relevant claudin-6 positivity and SWI/SNF gene alteration

**DOI:** 10.1038/s41598-024-64116-2

**Published:** 2024-06-12

**Authors:** Max Kraemer, Thomas Zander, Hakan Alakus, Reinhard Buettner, Su Ir Lyu, Adrian Georg Simon, Wolfgang Schroeder, Christiane J. Bruns, Alexander Quaas

**Affiliations:** 1 Faculty of Medicine, University Hospital of Cologne, Institute of Pathology, University of Cologne, Cologne, Germany; 2 Department of General, Visceral, Cancer and Transplantation Surgery, University Hospital Cologne, Cologne, Germany; 3 Department I of Internal Medicine, Center for Integrated Oncology Aachen Bonn Cologne Duesseldorf, Gastrointestinal Cancer Group Cologne GCGC, University of Cologne, Kerpener Str. 62, 50937 Cologne, Germany

**Keywords:** Fetal-gut, SALL4, Claudin-6, Glypican 3, SMARCA2-loss, Esophageal adenocarcinoma, Clear cell adenocarcinoma, Cancer screening, Tumour biomarkers, Gastrointestinal cancer, Oesophageal cancer, Cancer therapy, Drug development, Targeted therapies

## Abstract

Esophageal adenocarcinoma (EAC) is one of the deadliest tumor entities worldwide, with a 5-year survival rate of less than 25%. Unlike other tumor entities, personalized therapy options are rare, partly due to the lack of knowledge about specific subgroups. In this publication, we demonstrate a subgroup of patients with EAC in a large screening cohort of 826 patients, characterized by specific morphological and immunohistochemical features. This subgroup represents approximately 0.7% (6/826) of the total cohort. Morphological features of this subgroup show a striking clear cytoplasm of the tumour cells and the parallel existence of rare growth patterns like yolk sac-like differentiation and enteroblastic differentiation. Immunohistochemistry reveals expression of the fetal gut cell-like proteins Sal-like protein 4 (SALL4), claudin-6, and glypican 3. Interestingly, we find a correlation with alterations of SWI/SNF-complex associated genes, which are supposed to serve as tumor suppressor genes in various tumour entities. Our results suggest a possible implication of rare tumour subtypes in the WHO classification for EACs according to the classification for gastric cancer. Furthermore, claudin-6 positive tumors have shown promising efficacy of CAR T cell therapy in the recently published BNT-211-01 trial (NCT04503278). This represents a personalized therapeutic option for this tumor subtype.

## Introduction

Esophageal cancer is the 10th most common cancer worldwide in 2020 with 604.100 reported cases and has a five-year survival of less than 25%^1,2^. While the incidence of esophageal adenocarcinoma (EAC) is expected to increase within the upcoming years there is a simultaneous decrease of squamous cell carcinoma (ESCC)^1^. Although the focus of clinical research is shifting towards chemotherapy-free regimens, chemotherapy remains the backbone of EAC therapy in advanced tumor stages. Therefore, the identification of subgroups that can benefit from targeted and possibly less toxic treatment regimens is an important component of current research.

While the WHO classification of 2019 includes rare tumor entities for gastric carcinoma, such a classification does not exist for esophageal cancer^3^. These rare tumor entities include hepatoid adenocarcinomas as well as other alpha-fetoprotein (AFP)-producing tumours such as enteroblastic-differentiated adenocarcinomas and yolk-sac tumor-like carcinomas. More than one of these histological subtypes often coexist^3^.

Adenocarcinoma with enteroblastic differentiation show a tubulopapillary growth pattern and the carcinoma cells have a strikingly clear cytoplasm. Overall, the histology resembles that of a fetal intestine and fetal intestinal cell markers such as Sal-like protein 4 (SALL4), claudin-6, and glypican 3 are expressed at increased levels. Furthermore, an AFP expression may be present, although this is not mandatory^3^. There is limited clinical data available on gastric adenocarcinoma with enteroblastic differentiation. The largest study by Murakami et al. includes 29 patients and presents the morphological, immunohistochemical and clinical characteristics of this rare tumor entity^4^. In their patient cohort, there is a trend towards an aggressive tumor biology with a high rate of lymphatic and haematogenous metastasis^4^. Despite the prognostic significance of enteroblastic differentiation, the question arises whether this histological feature could even have therapeutic implications. Currently, e.g. targeted therapy options exist for solid tumours with claudin-6 expression, being evaluated in an open phase 1/2 trial (trial no.: BNT211-01).

Until now, nearly nothing is known about similar morphological features in esophageal cancer. Does this rare (gastral) tumor subtype also exist in the esophagus? If so, how common is it and what are the characteristics of these tumors in the esophagus? In this publication, we describe for the first time an enteroblastic differentiation with fetal gut cell-like cell markers in a group of patients with esophageal adenocarcinoma (EAC), analyse patients’ and tumor characteristics and discuss potential implications for clinical practice.

## Material and methods

### Patients and tumour samples

For screening our patient cohort for enteroblastic differentiation, we used the formalin-fixed and paraffin embedded samples from 826 patients with EACs. All patients underwent primary surgical resection or resection after neoadjuvant therapy between 1999 and 2017 at the Department of General, Visceral and Cancer Surgery, University of Cologne, Germany. The standard surgical procedures were laparotomic or laparoscopic gastrolysis and right transthoracic en-bloc esophagectomy, with intrathoracic esophagogastrostomy, including two-field lymphadenectomy of mediastinal and abdominal lymph nodes, transhiatal extended distal esophagectomy with intrathoracic or cervical anastomosis as described previously^5^. Preoperative chemoradiation (5-Fluouracil/cisplatin or carboplatin/paclitaxel + 40 Gy) or chemotherapy were administered in case of tumour stage ≥ c3.

For the screening procedure, we constructed a tissue microarray (TMA), as previously described^6^. In brief, for this TMA, we randomly punched out one tissue core from each tumour and transferred it into a TMA recipient block. Four μm sections of the resulting TMA blocks were transferred to an adhesive coated slide system (Instrumedics Inc., Hackensack, NJ) for immunohistochemistry (IHC).

The study protocol was in accordance with the ethical guidelines of the 1964 Declaration of Helsinki and its later amendments as reflected by the approval of the institution’s human research review committee (Ethics Committee of the Medical Faculty of University of Cologne: registration no. 13-091; Ethics-No. 21-1146). All patients gave written informed consent to the use of their tumour specimen and their data for research and publication.

### Immunohistochemistry

IHC was performed on the TMA slides and for positively screened cases on large-scale slides. The following antibodies were used for IHC studies: a mouse monoclonal antibody (clone 6E3; dilution 1:400; Cell Marque) for SALL4, a mouse monoclonal antibody (clone A-4; dilution 1:50; Santa Cruz/RUO) for claudin-6, a mouse monoclonal antibody (clone 1G12; dilution 1:50; Cell Marque/CE) for glypican 3, a mouse monoclonal antibody (clone A4; dilution 1:6000; Dako/CE) for cytokeratin 7 (CK7), a rabbit monoclonal antibody (clone D9E8B; dilution 1:50; Cellsignal/RUO) for SWI/SNF-related matrix-associated actin-dependent regulator of chromatin subfamily A member 2 (SMARCA2), a mouse monoclonal antibody (clone EPNCIR111A; dilution 1:300; abcam) for SWI/SNF-related matrix-associated actin-dependent regulator of chromatin subfamily A member 4, a rabbit monoclonal antibody (clone : EPR13501 dilution 1:1000; abcam) for AT-rich interactive domain-containing protein 1A (ARID1A), a monoclonal antibody (clone poly; dilution 1:1600; Dako/CE) for AFP. All immunohistochemical stainings were performed using the BOND-MAX-stainer (Leica Biosystems, Germany) according to the protocol of the manufacturer. The evaluation of immunohistochemical expression was assessed manually by two pathologists (A.Q. and A.G.S).

### Strategy of evaluation

We initially performed a screening for the expression of SALL4 (nucleus marker) and glypican 3 (surface marker) on the TMA. If at least one of the markers was detectable on more than 50% of the tumor cells, we analysed the case on a large-scale slide (whole tumor block). If the impression from the TMA that more than 50% of the tumor cells were positive for at least one of the two proteins (SALL4 or glypican 3) was confirmed on the large-scale slide, claudin-6 (surface marker) was additionally determined on whole tumor blocks. We provide the TMA data of the fetal-gut like cell markers SALL4, glypican 3, and claudin-6 in the supplementary material (Supplementary Table 1). Due to patients with low expression of fetal-gut like cell markers on the tumor cells, we have chosen a positivity cut-off of 50% in analogy to previous evaluation strategies of claudin-6 and glypican 3^7,8^. For further specification of a subgroup with enteroblastic differentiation, we focused on cases with the expression of at least 2 out of the 3 markers.

Cases showing an expression of at least two of the three markers claudin-6, SALL4 or glypican 3 on whole tumor slides were considered for further analysis. Following that, immunohistochemical stainings for CK7, SMARCA2, SMARCA4, ARID1A, and AFP were performed on the positively screened cases.

## Results

### Clinicopathological findings

The screening procedure identified 6 out of 826 patients (0.7%) who were positive for at least two out of the three markers SALL4, glypican3, or claudin-6. Five out of the six patients were male, average age at diagnosis was 63.5 (46–72). Also, five out of the six patients died within two years from diagnosis. The clinicopathological findings are summarized in Table 1.

**Table 1:** Baseline characteristics of the screening patient cohort.

	EAC total cohort (n = 826)	
	non-enteroblastic (n = 820; 99.3%)	enteroblastic (n = 6; 0.7%)
	n (%)	n (%)
Sex		
Male	717 (87.4)	5 (83.3)
Female	103 (12.6)	1 (16.7)
Median age (range)	64.0 (28–92)	63.5 (46–72)
(y)pT		
pT1	152 (18.5)	0 (0)
pT2	151 (18.4)	1 (25.0)
pT3	489 (59.6)	4 (50.0)
pT4	28 (3.5)	1 (25.0)
(y)pN		
pN0	333 (40.6)	0 (0)
pN+	487 (59.4)	6 (100.0)
Neoadjuvant treatment		
Yes	518 (63.2)	5 (83.3)
No	302 (36.8)	1 (16.7)

### Histological features

**Figure 1:**
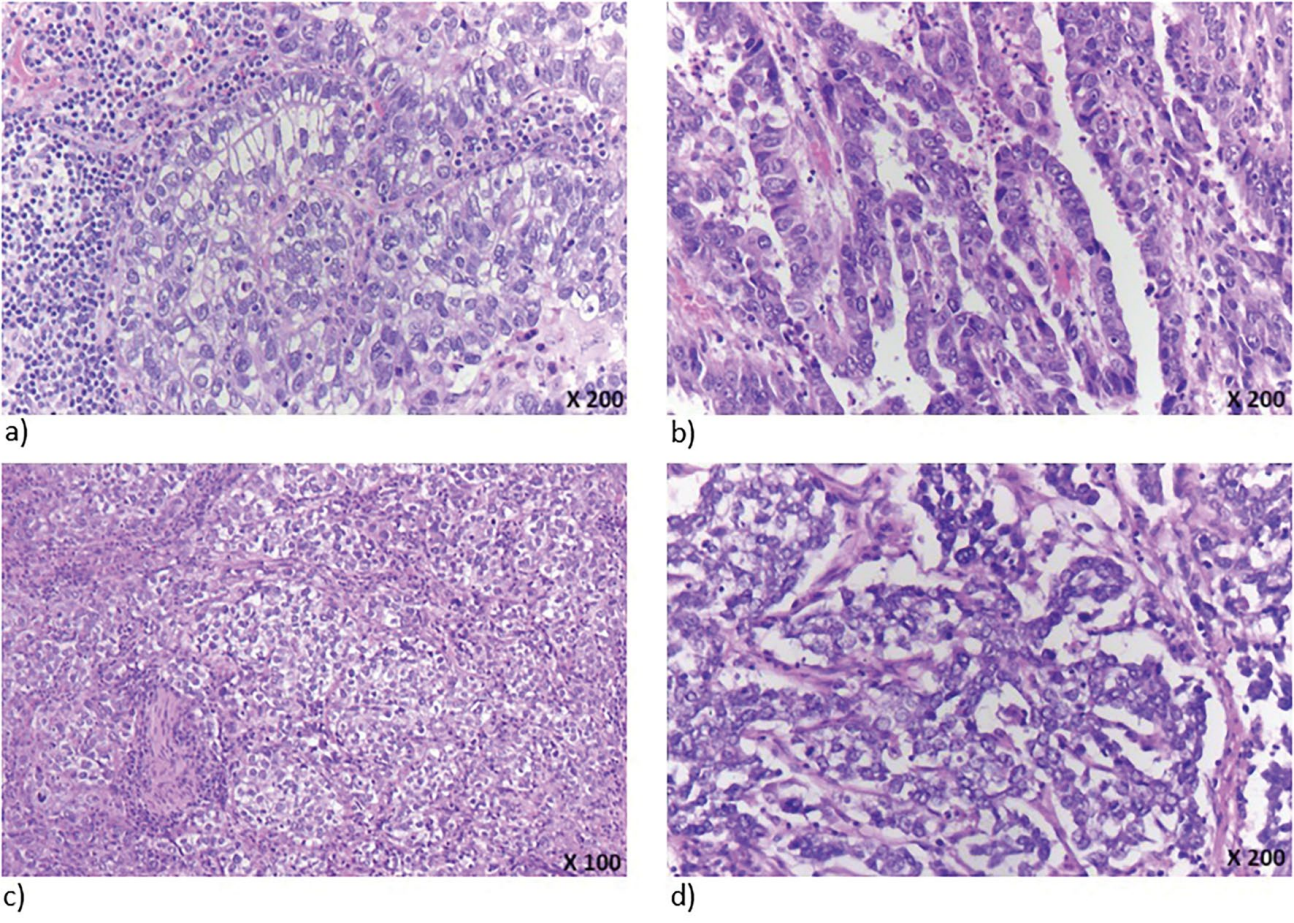
HE-morphology of different tumor growth patterns (different growth patterns can co-exist within the same tumor): (**a**) enteroblastic, (**b**) papillary, (**c**) solid clear cell, (**d**) garland “yolk-sac”-like.

All tumours showed parts with carcinoma cells with a strikingly clear cytoplasm. Very different tumor growth patterns were found. Only in one case the characteristics of enteroblastic differentiation were observed focally. In the remaining cases, tubulo-papillary or solid or garland-shaped growth patterns were detected, which suggested a yolk sac-like differentiation. These different growth patterns also occurred within the same tumor (Fig. 1).

### Immunohistological staining

**Figure 2:**
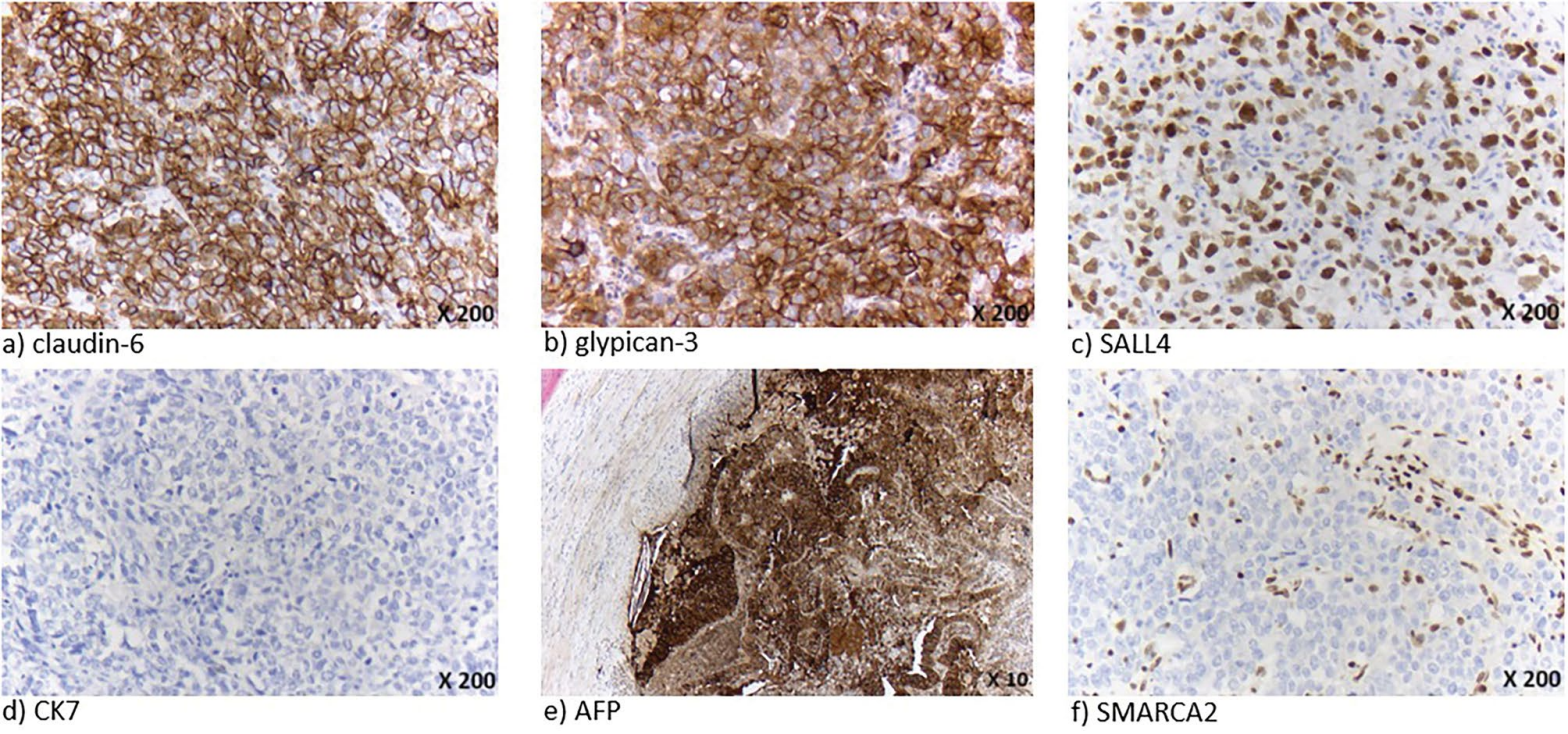
Immunohistochemistry in esophageal adenocarcinoma with expression of fetal-gut cell markers; (**a**) Claudin-6 expression on tumour cells, (**b**) glypican 3 expression on tumor cells, (**c**) SALL4 expression on the nucleus of the tumor cells, (**d**) CK7 loss of the tumour cells, (**e**) AFP expression on tumour cells (lymph node metastasis), (**f**) SMARCA2 loss of tumour cells (tumor nuclei in pale blue without expression of SMARCA2 protein and co-existing lymphocytes and other stroma cells within the tumor show preserved SMARCA2 expression).

The results of the performed immunohistological staining are summarized in Table 2. Regarding the markers of enteroblastic differentiation, all patients showed a claudin-6 and glypican 3 on at least 70% of the carcinoma cells (Fig. 2a,b). The SALL4 expression was heterogeneous, with three patients showing expression on at least 80% of the carcinoma cells (Fig. 2c). If positive, glypican 3, SALL4 and claudin-6 were ubiquitously expressed in the carcinoma cells throughout different growth patterns. Furthermore, all tumours showed marked reduced expression or absence of CK7 (Fig. 2d), various expression of AFP (Fig. 2e) and a poorly developed desmoplastic stromal reaction. Additionally, all tumours exhibited an alteration of at least one SWI/SNF marker, mostly a SMARCA2 loss (Fig. 2f).

**Table 2:** Immunohistological Staining results of the positively screened patient cohort.

Case	1	2	3	4	5	6
Sex	M	M	M	F	M	M
Age	46	63	66	72	66	68
CK7	2%	0%	5%	15%	0%	5%
SMARCA4	100%	100%	100%	100%	100%	100%
ARID1a	0%	100%	100%	100%	100%	100%
SMARCA2	0%	0%	10%	0%	10%	15%
AFP	2%	10%	0%	10%	90%	0%
Claudin-6	85%	100%	80%	100%	70%	90%
Glypican 3	70%	100%	80%	100%	85%	80%
SALL4	15%	80%	5%	100%	40%	10%

## Discussion

In this retrospective analysis of 826 patients with esophageal adenocarcinomas (EAC), we can identify a subgroup of patients characterized by the expression of fetal gut-like markers (glypican 3, SALL4, claudin-6). These patients exhibit not only immunohistochemical peculiarities but also morphological characteristics like a strikingly clear cytoplasm and loss or marked reduced CK7 expression. So far, there have only been case reports for this phenotype in EAC, making us the first to demonstrate these morphological and immunohistochemical characteristics in a group of patients within a large and representative patient cohort^9–11^.

With a frequency of 0.7%, we find the above-described characteristics only in a small portion of patients. In the WHO classification 2019 for gastric carcinomas, the frequency of AFP-producing carcinomas, including adenocarcinomas with enteroblastic differentiation, is estimated to be 0.3–2%^3^. The observed frequency in our patient cohort of EACs seems to be comparable to this. Despite this rareness, we know from other tumor entities like non-small cell lung cancer (NSCLC) that the identification of small sub-entities (like ROS1 altered NSCLC) can have a significant impact on the treatment concepts for the concerned patients^12^. It must further be mentioned that screening with TMA has the limitation that positive cases may not be detected due to intratumoral heterogeneity. We have still decided for using a high cutoff for positivity of protein expression (50%) to rather minimize the risk of false positive cases to select a homogenous and possibly biologically relevant subgroup.

In terms of therapeutic relevance, the consistent expression of claudin-6 in the identified subgroup could lead to such implications. Claudin-6 is a tight junction protein and is expressed to varying degrees in different tumor entities. For example, testis cancer shows increased expression in over 90% of tumor samples, while the rate in gastric carcinoma is around 3%^13^. Claudin-6 is the target protein for new therapeutic approaches, as for example a Chimeric Antigen Receptor T-Cell Therapy (CAR-T-cell therapy), which is currently being investigated in an early phase study by BioNTech (trial no. BNT-211-01). This therapy offers patients with solid tumours and a claudin-6 expression on > 50% of the tumor cells a new therapeutic option after the failure of approved therapy lines. In addition to claudin-6, also glypican 3 is currently being discussed in the context of targeted therapies for solid tumours. Monoclonal and bispecific antibodies, as well as CAR-T cell therapies targeting glypican 3 are being investigated in (pre-)clinical studies^14^. Until today, glypican 3 is mainly discussed in the context of hepatocellular carcinoma^15^. In gastric adenocarcinoma, glypican 3 expression has been described in tumours consisting of clear cytoplasm, which is totally in line with our findings, but there are no ongoing clinical trials targeting glypican 3 in this tumour entity^7^. For EAC, it seems reasonable that in the presence of an enteroblastic differentiation or a population with strikingly clear tumour cell cytoplasm in the haematoxylin and eosin staining or and loss/marked reduced CK7 expression on tumor cells, pathologists should consider the presence of fetal gut-like proteins and perform corresponding staining. Hereby, a subgroup of patients could significantly benefit from new therapeutic approaches.

Interestingly, we find a loss of SMARCA2 expression simultaneously to the expression of fetal-gut like cell markers in all patients. One patient additionally showed a loss of ARID1A expression. SMARCA2 and ARID1A are subunits of the SWI/SNF chromatin-remodelling complex, which is altered in up to 25% of all tumour entities^16^. In EAC, TCGA data describe SWI/SNF alteration across all subunits in approximately 20%, loss of function alterations of SMARCA2 in 4% and ARID1A in 8%^17,18^. At protein level, Schallenberg et al. report a loss of SMARCA2 expression in 9.9% and ARID1A in 10.4% in EAC^19^. However, an underlying oncogenic mechanism of SWI/SNF alteration remains unclear. Currently, an increased prevalence of genetic instability resulting from impaired DNA repair mechanisms, lineage-specific, epigenetic modifications and an association with distinct, oncogenic signalling pathways are being discussed^20–25^. Various early phase clinical trials are currently investigating the therapeutic potential of the suspected effects of SWI/SNF alterations, for example, by using poly(ADP-ribose)-polymerase (PARP) inhibitors in order to address impaired DNA repair mechanisms or Enhancer of zeste homolog 2 (EZH2) inhibitors to modulate certain epigenetic signatures^16^.

Furthermore, there is evidence for a particular role of the SWI/SNF complex in the differentiation of pluripotent embryonic stem cells^26,27^. In this context, the embryonic SWI/SNF complex prevents further differentiation of the stem cells by modifying the regulatory elements of the master pluripotency factors Octamer binding transcription factor 4 (Oct4), SRY-Box Transcription Factor 2 (Sox2), and Nanog^27^. The composition of the embryonic SWI/SNF complex is characterized by the absence of the ATPase SMARCA2, which is fully compensated by SMARCA4 expression^26,28^. The identified six patients with expression of fetal gut-like cell markers all show an alteration of SMARCA2 and, with the exception of one patient, a strong, preserved SMARCA4 expression. This constellation may also indicate the “fetal nature” (so called stem cell-derived differentiation) of the tumor cells. However, it remains unclear to what extent the differentiation stage of tumor cells has clinical relevance. Therefore, for now, rather the distinct characteristics of fetal differentiation, such as the expression of claudin-6 or abnormalities in the SWI/SNF complex, may potentially serve as targets for future therapeutic concepts.

## Conclusion

In conclusion, in our analysis, we identified a small subgroup of patients with EAC who exhibit morphological and immunohistochemical features of a fetal-gut like differentiation. For the clear distinction between tubular/papillary EACs composed of clear cells and EACs with an enteroblastic differentiation, we propose the following definition: Enteroblastic carcinomas show significant expression of at least two of the three fetal gut proteins (SALL4, glypican 3 and claudin 6) in their tumor cells. Significant means that at least 50% of the carcinoma cells must produce these proteins. There is often an absence/significant reduction of cytokeratin 7 (CK7), which is usually produced by adenocarcinomas of the esophagus with tubulo-papillary morphology. Due to the limited number of cases, the clinical characteristics of these patients are still unknown, and the 2019 WHO classification does not separately list this subgroup of esophageal carcinoma. However, increased attention to the described features in routine diagnostics may help identify more patients with this phenotype and contribute to further characterization. There is already today a therapeutic relevance for this subtype, suggesting that it could have significant importance in the future.

### Supplementary Information

Supplementary Tables.

## Data Availability

The datasets generated during and/or analysed during the current study are available from the corresponding author on reasonable request.
